# EWORS: using a syndromic-based surveillance tool for disease outbreak detection in Indonesia

**DOI:** 10.1186/1753-6561-2-s3-s3

**Published:** 2008-11-14

**Authors:** Hadi Siswoyo, Meda Permana, Ria P Larasati, Jeffryman Farid, Asep Suryadi, Endang R Sedyaningsih

**Affiliations:** 1 Biomedic and Pharmacy Research Center, National Institute of Health Research and Development, Indonesian Ministry of Health, Jakarta, Indonesia; 2 U.S. Naval Medical Research Unit No. 2 (NAMRU-2), Jakarta, Indonesia

## Abstract

**Background:**

Electronic syndromic surveillance for early outbreak detection may be a simple, effective tool to rapidly bring reliable and actionable outbreak data to the attention of public health authorities in the developing world.

**Methods:**

Twenty-nine signs and symptoms from patients with conditions compatible with infectious diseases are collected from selected Provincial hospitals and analyzed daily. Data is e-mailed on a daily basis to a central data management and analysis center. Automated data analysis may be viewed at the hospital or the Early Warning Outbreak Response System (EWORS) hub at the central level (National Institute of Health Research and Development/NIHRD).

**Conclusion:**

The Indonesian Ministry of Health (MoH) has adopted EWORS since 2006 and will use it as a complementary surveillance tool in wider catchment areas throughout the country. Socialization to more users is still being conducted under collaboration of three Directorate Generals (DGs) of the MoH; DG of NIHRD, DG of Medical Services and DG of Communicable Disease Control and Prevention. Currently, EWORS is being adapted to facilitate detecting a potential outbreak of pandemic influenza in the region, and automated procedures for outbreak detection have been added.

## Background

**Figure 1:**
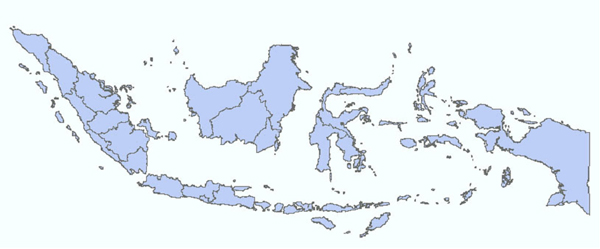
The Republic of Indonesia.

Indonesia is the largest archipelago with approximately 17,700 islands (Figure 1). It is a developing country with 33 provinces and a population of about 230 million people. Like other developing countries, Indonesia experiences substantial episodes of infectious disease outbreaks each year. Its tropical climate, dense population (in some islands), and poor health infrastructure provide a fertile ground for emerging and re-emerging infectious diseases. Lately, Indonesia has been suffering from human infection of Influenza A (H5N1). The Case Fatality proportion was the highest in the world [unpublished data]. This new health problem appeared while outbreak of some "old" diseases, such as Dengue Hemorrhagic Fever (DHF), is still seen during the rainy seasons.

An electronic syndromic surveillance for early outbreak detection may be a simple, effective tool to rapidly bring reliable and actionable outbreak data to the attention of public health authorities in the developing world. This paper describes the experience of using such a system in Indonesia.

The Early Warning Outbreak Recognition System (EWORS) in Indonesia is a hospital-based network of computerized linkages that provides syndromic surveillance for early detection of infectious disease outbreaks. It establishes trend information that helps to distinguish epidemic from endemic disease activity. The objective of the EWORS program is to get information about infectious disease activity more quickly, and thus allow more timely public health interventions [1].

The EWORS program was developed in 1998 by the U.S. Naval Medical Research Unit #2 (NAMRU-2) and the National Institute of Health Research and Development (NIHRD) (*LitBangKes*) of the Indonesian Ministry of Health (MoH). The EWORS program complements the existing reportable disease surveillance conducted by the Surveillance Directorate of the Indonesian CDC, which uses a manual, paper-based system to collect data from Provincial and District Health Offices.

Since January 1999, NAMRU-2 (Jakarta, Indonesia) has trained 35 sites in five countries (Indonesia, Vietnam, Cambodia, Laos, and South Korea) to use the EWORS program. Worldwide, EWORS is now available in six different languages (English, Vietnamese, Laotian, Korean, Chinese and Spanish).

The symptom- and syndrome-oriented approach makes EWORS capable of detecting outbreaks of new diseases, including man-made biological threats. Currently, EWORS is being adapted to facilitate the detection of potential initial outbreaks of pandemic influenza in the region. Automated procedures for outbreak detection have also been added.

## Methods

We selected a number of major public hospitals in some provinces as EWORS sentinel surveillance sites, mostly located in areas with substantial epidemic potential. Twenty-nine signs and symptoms, as well as demographic data, e.g., age, sex, address, are collected on a daily basis from patients visiting a pediatric clinic, internal medicine or emergency room with conditions compatible with a suspected infectious disease and recorded on a standardized form. At the end of each day, the forms are submitted to the EWORS operator for data entry into the system. Data is transmitted on a daily basis via e-mail to the central data management and analysis center (hub) at the National Institute of Health Research and Development, Ministry of Health and stored securely both in the hospital computer's hard drive as well as in the SQL server at the data center. Data may be analyzed at the hospital or the hub for case count trends over time, as well as demographic trends in abnormal increases of cases with clinical signs and symptoms consistent with a syndrome, such as watery diarrhea, that could reflect a *Vibrio cholera *infection or hemorrhagic manifestation suggestive of dengue virus infection (dengue hemorrhagic fever). Additionally, the EWORS's symptom-oriented approach makes it capable of detecting outbreaks of new diseases, including man-made biological threats. Intensive on-site training and subsequent upgrading and re-training of each hospital's staff are conducted to ensure standardization of data collection and analysis within and between sites.

## Results

In 1999, EWORS was implemented in 11 hospitals in 8 provinces. In 2008, only 9 hospitals in 7 provinces continued to participate in the program (Table 1). Twenty-nine signs and symptoms compatible with infectious diseases of patients visiting the surveillance sites were collected and analyzed daily (Table 2). Data was emailed on a daily basis to a central data management and analysis center at the EWORS hub at NIHRD, Jakarta. Automated data analysis could also be viewed at the site hospitals.

**Table 1:** The number of provincial hospitals who have implemented the EWORS program

**No.**	**Hospital Name**	**Province**	**Date Implemented**
1	Sanglah, Denpasar	Bali	October 1998
2	Labuang Baji, Makasar	South Sulawesi	February 1999
3	Soedarso, Pontianak	West Kalimantan	March 1999
4	Pirngadi, Medan,	North Sumatera	April 1999
5	Persahabatan, Jakarta	Jakarta	September 1999
6	Sulianti Saroso, Jakarta	Jakarta	May 2000
7	Sarjito, Yogyakarta	Yogyakarta	December 2000
8	AW Sjachranie	East Kalimantan	March 2003
9	Kanujoso, Balikapapan	East Kalimantan	March 2003

**Table 2:** List of signs/symptoms

**No.**	**Signs/Symptoms**	**No.**	**Signs/Symptoms**
1	Abdominal discomfort	16	Fever
2	Anuria/oliguria	17	Headache
3	Bloody cough	18	Hematemesis/melena
4	Bloody diarrhea	19	Jaundice
5	Bone/muscle/joint pain	20	Malaise
6	Bubo-lymphadenitis	21	Mental status disturbances
7	Chills	22	Nausea
8	Common cold	23	Paralysis
9	Conjunctivitis	24	Rash
10	Cough	25	Seizures
11	Cutaneous bleeding	26	Sore throat
12	Dark urine	27	Stiff neck
13	Dehydration	28	Vesicle/bullae
14	Diarrhea	29	Vomiting
15	Difficult breathing		

**Figure 2:**
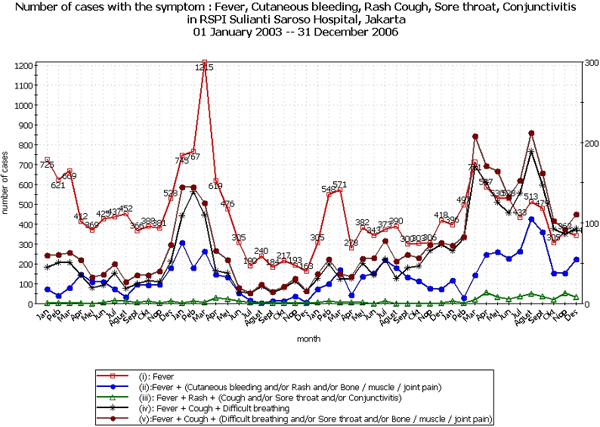
Number of cases with the symptom: fever, cutaneous bleeding, rash cough, sore throat, conjunctivitis in RSPI Sulianti Saroso Hospital, Jakarta, 01 January 2003 – 31 December 2006.

Two examples of how the system worked are described here. In the first example, EWORS detected a large DHF outbreak in 2003 that showed a moving trend across Indonesia from west to east. It started in Medan (Sumatera Island) in the west, spread to Jakarta (Java Island) and then to Bali Island. It also spread to Makassar, Sulawesi Province, in the north. What we observed was an excess of fever hemorrhagic cases starting in Medan in November 2003 (Figure 2), followed a month later by excess of similar cases in Jakarta in December 2003. A month later, an excess of fever hemorrhagic cases were noted in Bali, and in February 2004, excess cases were detected in Makassar. In the second example, EWORS was also able to detect a Leptospirosis outbreak (a combination of fever and jaundice) in Jakarta in 2006.

## Discussion and recommendations

The EWORS program in Indonesia has experienced many challenges, particularly the high cost to continue the program, difficulties in creating team work, and struggling to maintain enthusiasm and commitment by participating hospitals. In our experience, successful implementation and continuation of the EWORS program in a hospital will depend on human resources, hospital commitment, the EWORS package, finances, computer networking, technical aspects, and data analysis results.

We are convinced that to be able to effectively implement the EWORS Program, a hospital needs the following human resources:

• A hospital program manager who is an epidemiologist or a person who likes working with statistics or data analysis.

• A data entry clerk who is dedicated exclusively to entering data for the EWORS program. This is the heart of the system's success. Using a clerk who has other duties has not been successful.

• Physicians and nurses in the emergency room, pediatric clinic, and internal medicine clinic to maintain continuity and train new physicians and nurses in documenting information for the EWORS cleark. Training and re-training of physicians and nurses should be conducted on a regular basis.

Each hospital director must be committed to the EWORS program to create hospital policies that support the system. One possible benefit to the hospital is the early warning of increased workload due to an outbreak. To further increase support from hospitals, we have been trying to embed the EWORS Program in the MoH system. We have invited the directorate general in charge of medical services to co-sponsor the EWORS system along with the NIHRD and the Indonesian CDC.

This program requires information technology support, such as special phone lines, dedicated computers, as well as suitable data collection forms in the emergency room/internal medical room/pediatrician room for each patient visit. The team (doctor, nurse, data entry clerk) must be well trained and have delegation of authority to continue this program. The data entry clerk should have suitable work space, ideally a room dedicated to EWORS.

The program manager must be able to analyze the data in a manner that is clear, standardized, and useful for public health control measures. The analysis should be more frequent during an outbreak. This program needs team work among all the people who make the system work, from the hospital to the provincial or district health officer. The delivery system of the data can be day by day.

Discussions are currently underway about expanding the EWORS system to all provincial hospitals in Indonesia in 2008. We are also considering including private hospitals in the EWORS program. We would like to give a Quality Award to hospitals who properly maintain the EWORS program. We have improved guidance on data analysis in 2007. In 2008, we plan to study EWORS data using a sensitivity test in nine hospitals, so we can compare EWORS data with routine surveillance data collected at the district health office.

## Conclusion

The EWORS program was developed to complement the existing disease surveillance and provide a simple, flexible surveillance system that detects disease outbreaks early after their onset. The program complements the existing reportable disease surveillance conducted by the Surveillance Directorate of the Indonesian CDC, which uses a manual, paper-based system to collect data from Provincial and District Health Offices. The program's success is dependent on many factors such as human resources, hospital commitment, computer networking, and technical expertise. Because of limited clinical laboratory data in many developing countries, widely implementing the EWORS syndromic surveillance system may allow for more rapid mapping of infectious disease outbreaks and aid control efforts.

Currently, EWORS is being adapted to facilitate detection of a potential outbreak of pandemic influenza in the region, and automated procedures for outbreak detection have been added.

## Competing interests

The authors declare that they have no competing interests.

## Authors' contributions

The corresponding author is the chief coordinator of EWORS activities in Indonesia.
